# The Role of Information and Communication Technologies in Clinical Trials with Patients with Alzheimer’s Disease and Related Disorders

**DOI:** 10.3389/fnagi.2015.00110

**Published:** 2015-06-09

**Authors:** Alexandra König, Guillaume Sacco, Gregory Bensadoun, Francois Bremond, Renaud David, Frans Verhey, Pauline Aalten, Philippe Robert, Valeria Manera

**Affiliations:** ^1^CoBTeK Cognition Behaviour Technology EA 7276, Research Center Edmond and Lily Safra, University of Nice Sophia Antipolis, Nice, France; ^2^School for Mental Health and Neuroscience, Alzheimer Center Limburg, Maastricht University Medical Center, Maastricht, Netherlands; ^3^Rehabilitation Unit, Department of Geriatrics, CHU de Nice, Nice, France; ^4^Centre d’Innovation et d’Usages en Santé (CIU-S), Cimiez Hospital, University Hospital of Nice, Nice, France; ^5^STARS, INRIA, Sophia Antipolis, France; ^6^Centre Mémoire de Ressources et de Recherche, CHU de Nice, Nice, France

**Keywords:** clinical trials, Alzheimer’s disease, mild cognitive impairment, Information and communication technologies (ICT), sensors, outcome measures, endpoints, assessment tools

## Introduction

In the last decades, many promising disease-modifying treatments for Alzheimer’s disease (AD) have been proposed. However, clinical trials conducted on the treatments’ efficacy have not lead to any important breakthroughs. There is a growing consensus that this can, at least partially, be explained by methodological difficulties, including the inclusion of participants who are already in the later stages of the disease progression, and the selection of outcome measures – such as dementia conversion rate – which are not sensitive enough (Aisen et al., 2011).

Most of the current assessment tools have been accused to be artificial and to lack ecological validity (Robert et al., 2013). Furthermore, test results can show variability depending on many factors, such as the patient’s emotional state, and may therefore not always fully reflect a patient’s capacities and the complexity of the disease, leading to delayed diagnosis (Sampaio, 2007).

Based on the Monaco CTAD expert meeting in 2012, Robert et al. (2013) highlighted that new Information and Communication Technologies (ICT) – such as video and audio analysis techniques, computerized testing and actigraphy – may represent promising new tools to improve the functional and cognitive assessment of patients with Alzheimer’s disease (AD) and related disorders [see also Konig et al. (2014), for a recent review of studies employing ICT in this domain]. However, these new technologies are still not widely employed in clinical trials for assessment purposes. In November 2014, the association Innovation Alzheimer organized a workshop with stakeholders in the field (e.g., psychiatrist, neurologists, geriatricians, psychologists, researchers, engineers, and patients) with the aim of gathering recommendations for the use of ICT in the different stages of clinical trials. These recommendations are available online on the website of the Association Innovation Alzheimer^1^.

Based on these recommendations, in the present opinion paper, we will highlight how ICT may be employed in clinical trials involving patients with AD and related disorders to improve patient’s assessment and the admissibility to participate in clinical trials.

## The Current Use of ICT in Clinical Trials

Information and Communication Technologies is now widely employed in several stages of clinical trials. For instance, pharmaceutical companies and Contract Research Organizations routinely adopt E-trainings for investigators. Patients’ recruitment can take advantage of the wide employ of Electronic Health Records storing health-related data (Hsiao and Hing, 2014), and E-recruitment methods employing social media and the Internet are also starting to emerge. Similarly, data entry is now facilitated by electronic Case Report Forms, employed in almost the totality of the clinical trials leaded by pharmaceutical companies (Kuchinke et al., 2010). However, ICT is still not consistently used in clinical trials at the assessment stage.

ClinicalTrials.gov – a registry and results database of publicly and privately supported clinical studies of human participants conducted around the world – contains at present (January 2015) more than 2500 clinical trials involving participants with mild cognitive impairment (MCI), AD, or other dementia types. We performed a keyword-based^2^ search on these trials focusing on automated audio and video analysis techniques, actigraphy, and computerized testing. Only 16 pharmaceutical trials employing ICT for assessment purposes were retrieved: 6 employing accelerometers and 10 employing computerized testing. No study employing automated audio or video analysis techniques was found. While it is certainly possible that these numbers represent an underestimate, they suggest that more work should be done to bring the clinical domain closer to the frontiers of the clinical research.

## ICT for Assessment in Clinical Research

**Figure 1:**
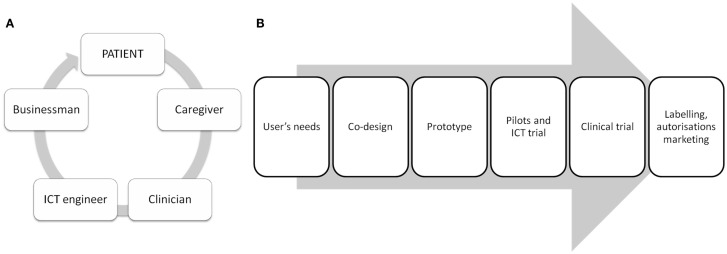
**Flowchart representing the different stakeholders (A) and the main steps involved in the design of ICT solutions for the health domain (B)**. **(B)** User’s needs: finding and screening the patient’s needs with patients, caregivers, and clinicians. Co-design: generating ideas and selecting viable ICT solutions with patients, caregivers, clinicians, and ICT engineers. Prototype development: developing a first ICT prototype with clinicians, ICT engineers, and businessmen. Pilot and ICT trial: initial tests on the usability/feasibility of the ICT solution, followed by prototype modifications. Clinical trial: study on a larger, well-defined patient’s population in order to test the efficacy of the ICT solution in short and medium terms. Labeling, authorizations, marketing: leaded by ICT/business stakeholders with the help of clinicians, patients, and caregivers.

The design of ICT solutions for the health domain is a complex process, which requires the close collaboration of different stakeholders (see Figure 1). Recent evidence suggests that ICT can play a crucial role in the assessment of AD and related disorders, both in terms of providing additional information for an earlier and more accurate diagnosis, and in terms of monitoring of the disease progression (Robert et al., 2013). For instance, it has been shown that automatic speech analysis techniques – analyses of verbal communication through computerized speech recognition interfaces – can represent a non-invasive and cheap method to gather information about verbal communication impairments, which are very common in patients with MCI and in the early stages of AD (Satt et al., 2013). These techniques are useful for automating the analysis of clinical and neuropsychological tests employed to assess linguistic abilities (such as verbal fluency and sentence repetition tests). But even more importantly, they can provide additional information that cannot be gathered in a clinical setting, such as utterance duration, filler typology, and analysis of voiced and voiceless segments. Recently, we showed that the vocal markers extracted from speech signal processing techniques differed significantly among healthy elderly participants, MCI, and early AD patients with accuracy higher than 80% (König et al., 2015a,b).

Similar observations apply to automatic video analysis techniques (Romdhane et al., 2012; Sacco et al., 2012; Konig et al., 2015c). These techniques have proven to be useful for fall detection and to improve home safety (Robinovitch et al., 2013), but recently they started to be adopted also for assessment purposes. For instance, in the FP7 project Dem@Care^3^ video analysis techniques are employed to provide objective measures to assess functional impairments in activities of daily living in elderly people and patients with MCI and AD. In the classical clinical settings, autonomy in activities such as taking medications, or handling finances are assessed through self-reports and informant-based questionnaires, which do not offer accurate, reproducible, objective, and ecological measures of functional performance. Using non-invasive 2D video recordings combined with video signal analysis, Konig et al. (2015c) showed that activities of daily living can be accurately detected and recognized by automated activity recognition algorithms, as suggested by results highly consistent with the clinician’s evaluation. Furthermore, video analysis allowed obtaining finer-grade measures, such as the time spent on each activity, which could not be captured in the classical clinical evaluation. Siraly et al. (2015) investigated if early signs of cognitive decline could be monitored by computer memory games with the results that healthy elderly subjects achieving lower scores in the memory game have increased level of atrophy in the temporal brain structures and showed a decreased performance in the Paired Associates Learning (PAL) test. Thus, computer games may be useful tools in early screening for cognitive decline. Similarly, online questionnaires tapping risk and protective factors in different health domains (e.g., diet, physical and cognitive activity, social engagement), such as those developed in the FP7 project InMINDD^4^, are starting to be employed to assess brain health and to screen for participants at risk of developing dementia.

A final example is represented by actigraphy, which is frequently used to monitor motor activity and rest-activity rhythms (Hatfield et al., 2004), and it has been proposed as an observer-independent evaluation method in different disorders, including dementia (Yakhia et al., 2014). Specifically, its utility as an assessment tool in AD and related disorders has been proven to assess neuropsychiatric symptoms such as agitation (Nagels et al., 2006; Mahlberg and Walther, 2007), depression (Volkers et al., 2003), and apathy (David et al., 2012). See Konig et al. (2014) for recent reviews on the use of actigraphy for assessment in patients with AD and related disorders.

## Why Should ICT be Employed More Consistently in Clinical Trials?

As detailed above, ICT-based techniques may represent non-invasive, objective, and inexpensive solutions to detect early cognitive and functional decline in patients with AD. Clinical interventional trials may take advantage of these solutions in several ways. First, ICT may contribute to determine the admissibility of participation in clinical trials at earlier stages of the disease, when treatment is supposed to be more effective. Patient’s performance scores on one assessment may fluctuate as a function of daily rhythms, fatigue, emotion, stress, and many other state-dependent factors. Due to this variance, certain difficulties present in the earliest stages of AD and related disorders may be undetectable during the classical assessment. ICT may be of great interest in this respect, because they enable the patients’ performance to be captured and accurately evaluated in real time and real life situations, even at the patient’s home (Robert et al., 2013). Second, ICT may help in providing a more timely conversion diagnosis, thus improving the sensitivity of outcome measures based on conversion rate as end-point of the intervention. Similarly, by allowing easy and non-invasive continuous monitoring of the patient over time, ICT can help assessing subtle changes in behavioral, cognitive, and functional patterns, and thus contribute to the definition of outcome measures finer than dementia progression or neuropsychological test scores. Finally, ICT may provide an interesting solution for remote assessment and follow-up. One of the challenges faced by big cohort clinical studies is that there is a consistent drop-out rate, at least partially due to the fact that patients need to go to a clinic for the assessments and follow-ups. ICT solutions combined with safe data transfer methods may reduce drastically the number of required visits, thus reducing the drop-out rate and the costs/time associated with the clinical trial.

An interesting example of how ICT could be employed in clinical trials is represented by the assessment of agitation. Agitation represents one of the most frequent neuropsychiatric symptoms in patients with dementia, and one of the most challenging symptoms to manage for primary caregivers (Okura and Langa, 2011). Following the Agitation Definition Work Group provisional consensus definition (Cummings et al., 2015), agitation in patients with cognitive disorders is defined by (A) the presence of criteria for a cognitive impairment or dementia syndrome, and (B) the presence at least one of the following behaviors associated with observed or inferred evidence of emotional distress for a minimum of 2 weeks, which represent a change from the patient’s usual behavior: (a) excessive motor activity; (b) verbal aggression; (c) physical aggression.

As for cognition, pharmacological solutions for agitation have given so far disappointing results (Soto et al., 2014). However, recently a new promising treatment has been released and tested, and showed preliminary efficacy evidence in larger cohort trials (Cummings et al., 2014; Siffert, 2014). ICT could play a key role in assessing agitation in patients with AD, and to test the new treatment efficacy. For instance, accelerometers could be employed to measure objectively the presence of abnormal motor activity. Speech analyses that extract automatically vocal features of recorded speech could be employed to assess verbal aggression in a more subtle and objective way. Finally, automated video analysis and activity-recognition techniques may be useful to quantify the appearance of certain activities and movement sequences that underline physical aggression.

## Conclusion and Future Research Directions

In order to progress in the validation of the treatments for AD, better outcome measures for cognitive and functional changes are acutely needed in the earliest stages of the pathology (Snyder et al., 2014). The clinical assessment of cognitive and functional changes in AD has traditionally relied on cognitive screening tests that are not always sensitive to the earliest cognitive, functional, and behavioral changes important to detect for effective preventive interventions (Snyder et al., 2014), are possibly subjected to variations in the clinical interpretation, and are not always good predictor of the progression from MCI to AD (Schmand et al., 2012). Furthermore, current diagnostic measures can be invasive (CSF analyses), expensive (neuroimaging), time-consuming (neuropsychological assessment), and are often available only in specialized clinics, which lead to reduced accessibility as frontline screening tool for AD and related disorders (Laske et al., 2014). Therefore, we face an increasing need for additional population-based screening and follow-up instruments with simpler and timelier adapted, non-invasive, and cost-effective tools allowing early identification of subjects in preclinical stages of AD.

Here, we highlighted how new tools involving ICT may represent an optimal solution to most of these challenges. However, in order to successfully integrate ICT measurements into clinical trials, some work has still to be done (Robert et al., 2013, 2014). Specifically, the use of such technologies should be validated in larger cohorts to demonstrate their clinical meaningfulness by correlating with available clinical diagnostics and biomarkers and thus receive recognition in the clinical scientific and medical world. Importantly, in addition, the use of ICT in clinical trials needs to be validated by Health authorities and policy makers. On the technological side, work in terms of system development and sensors integration has to be carried out to allow a reliable and complete assessment of a patient by merging information coming from different sensors into easily understandable feedback. The immediate and accurate visualization of the recorded data is of great importance to facilitate an easy use in clinical practice and to provide feedback to patients and their caregivers.

## Conflict of Interest Statement

The authors declare that the research was conducted in the absence of any commercial or financial relationships that could be constructed as a potential conflict of interest.

## Footnotes

^1^http://www.innovation-alzheimer.fr/homepage/

^2^Keywords : “audio analysis”, “speech analysis”, “audio recording”, “voice recording”, “voice recognition”, “video recording”, “video recognition”, “video analysis”, “3D recognition”, “accelerometer”, “actigraph” “actigraphy”, “motion sensor”, “computerized test/testing”, “computer test/testing”

^3^www.demcare.eu

^4^http://www.inmindd.eu/
